# Chemical Reactivity Parameters to Analyze Psychedelics: How Do We Explain the Potency of the Drugs?

**DOI:** 10.1021/acsomega.4c05726

**Published:** 2024-09-11

**Authors:** Ana Martínez, Alexis Caballero, Rodrigo Ramírez, Emiliano Perez-Sanchez, Esperanza Quevedo, Diana Salvador-García

**Affiliations:** Departamento de Materiales de Baja Dimensionalidad, Instituto de Investigaciones en Materiales, Universidad Nacional Autónoma de México, Circuito Exterior S. N. Ciudad Universitaria, CDMX, Mexico CP 04510, Mexico

## Abstract

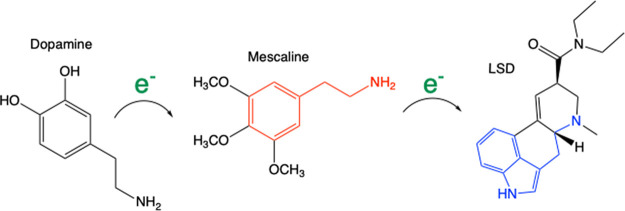

Psychedelics are psychoactive substances that produce changes in thoughts and feelings and modifications in perceptions of reality. The most potent psychedelic is also the first semisynthetic hallucinogen (lysergic acid diethylamide). Psychedelics have been investigated for decades because of their potential therapeutic effects in the treatment of neuropsychiatric diseases and also because these drugs are useful in controlling addictions to other substances. In this investigation, we analyze 27 psychedelic molecules. These compounds are serotonergic psychedelics; that is, they are serotonin agonists. We analyze the electron transfer properties to better understand the mechanism of action of these substances. We found that the electron acceptance capacity is related to the potency of the drugs: the best electron acceptor is also the most potent drug. We also used global softness as a parameter of reactivity. Molecules with greater global softness are more polarizable and also have greater potency. These results are useful to continue our understanding of the mechanism of action of psychotropic drugs.

## Introduction

Psychedelics or hallucinogens are psychoactive substances that stand out for their ability to cause a wide range of effects on consciousness, as well as changes in thoughts, feelings, and perceptions of reality.^1−11^ Hallucinogens derived from plants have been used for millennia by different cultures in ceremonial and social practices. The first semisynthetic hallucinogen [(lysergic acid diethylamide (LSD)] was obtained by Albert Hofmann in 1938.^1,4^ After having accidental physical contact with LSD, Hofmann described the experience as “an uninterrupted stream of fantastic pictures, extraordinary shapes with intense, kaleidoscopic play of colors”. This discovery marked a period of intense scientific research on the subjective effects caused by these substances, trying to elucidate their mechanism of action in the brain and their therapeutic potential.^1−4^ In 1947, LSD was first used as a psychiatric medication and to study the nature of psychoses.^1^ Almost all hallucinogens are illegal, and researchers do not consider any amount of use to be safe. However, in small quantities for specific cases, it has been used in psychedelic-assisted psychotherapy.^12−20^

Psychedelic-assisted psychotherapy is an emerging therapeutic approach. It is a treatment for resistant depression that uses psychedelics and psychotherapy.^16,17^ Experiments in animal models and observations in humans have provided evidence that psychedelic effects are mediated by serotonin agonism at the (5-HT)_2A_ receptor.^2,21−27^ The serotonergic hypothesis of psychedelic action states that psychedelics cause their effects via a common mechanism based on agonism at this receptor. In spite of substantial variation in the chemical structure, the subjective effects induced by psychedelics can be considered similar. Some distinctions in the effects have been attributed to variations in dose, the internal state of the user (set), and the surrounding (setting).^6,7^ Receptor binding profiles may also explain the functional selectivity of different psychedelic molecules.^3^

**Figure 1:**
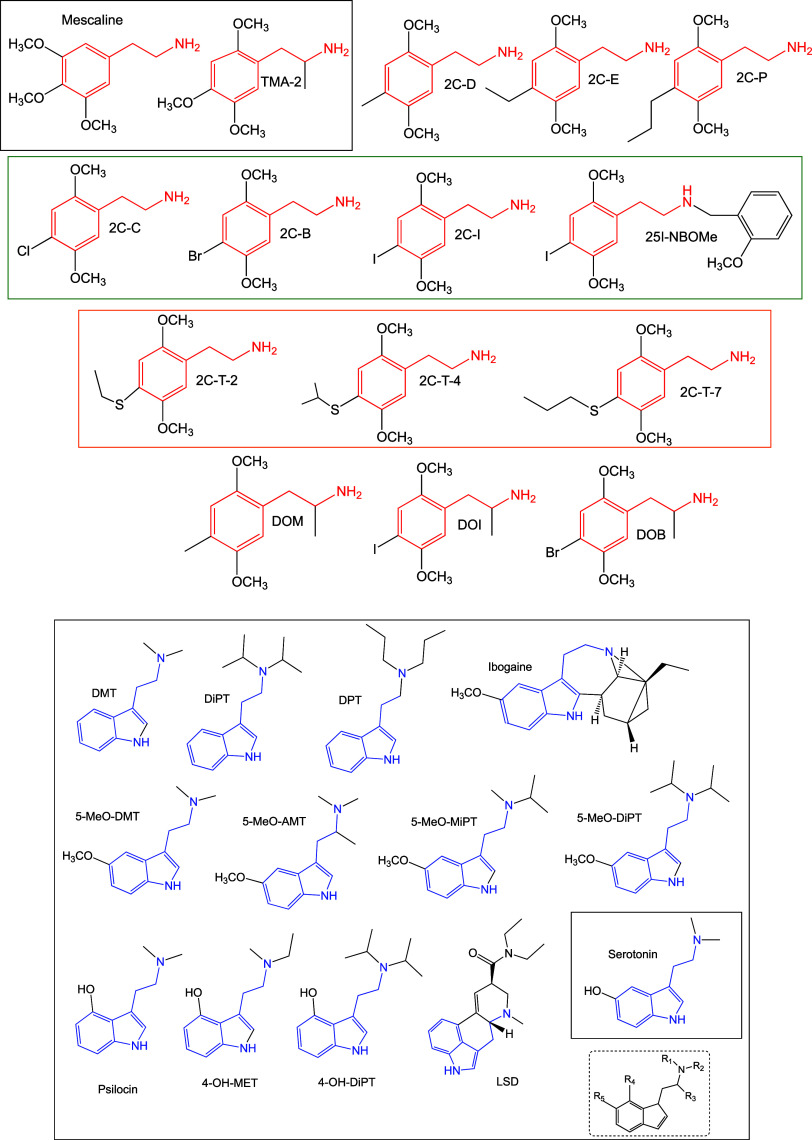
Molecular structure of compounds under study.

Psychedelics have been investigated for decades due to their potential therapeutic effects in the treatment of neuropsychiatric diseases and also because these drugs are useful in managing addictions to other substances.^12−20^ Clinical trials have demonstrated the effectiveness of psychedelic use in relieving symptoms of depression and anxiety and in promoting substantial control in nicotine and alcohol use. While these studies provide evidence for the desirable use of psychedelics as therapeutic drugs, the underlying molecular reasons for the mechanisms of action are still not well understood. To contribute to the understanding of the reactivity of these molecules, in this investigation, we analyze the electron donor–acceptor capacity of 27 psychedelic molecules (see Figure 1). We used a model that was previously reported for the analysis of various systems, such as antipsychotic drugs and the drugs used to control the symptoms of panic and depressive disorders.^28−33^ In the preceding works, it was concluded that dopamine agonists are electron donors, just like dopamine, while most antagonists are electron acceptors. With this model, it was possible to propose a new characterization of antipsychotics based on electron transfer capacity.^29^ In the investigation presented here, we characterize psychedelic drugs in a similar way. Moreover, we also analyze global softness as a parameter of reactivity. Our results represent useful information to interpret the potency of drugs and to increase knowledge about the possible action mechanisms of psychedelics.

### Computational Details

Gaussian09 was used for all electronic calculations.^34^ Geometry optimizations of initial geometries were obtained at the wB97xd/6-311+g(2d,p) level of theory without symmetry constraints.^35−38^ This is the latest functional from Chai and Head-Gordon, which includes empirical dispersion and long-range corrections.^35^ LAN2DZ basis set was used for the compounds with I. Harmonic analyses were performed to verify local minima.

Conceptual density functional theory is a chemical reactivity theory found on density functional theory-based concepts.^39−43^ Within this theory, there are global response functions, such as the electro-donating (ω) and electro-accepting (ω+) powers, as previously reported by Gázquez et al.^40,41^ The capacity to donate electrons (ω−) and the propensity to accept electrons (ω+) are defined as follows:
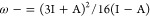
1
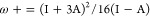
2where I and A are the vertical ionization energy and vertical electron affinity, respectively. They are obtained as follows:
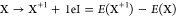
3
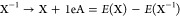
4

**Figure 2:**
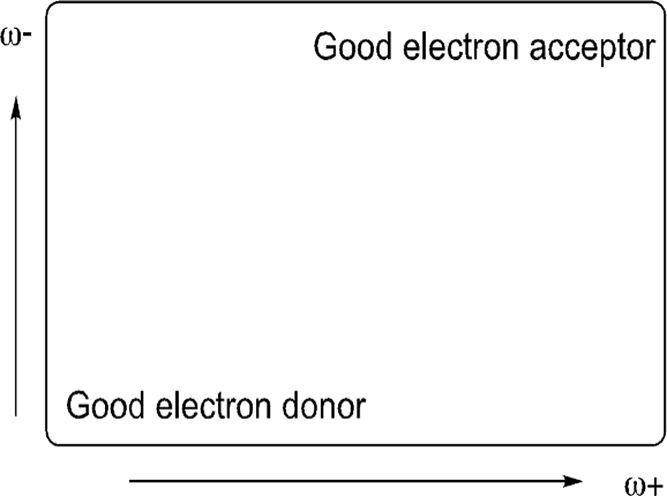
Electron DAM reproduced from ref (28). Copyright [2008] American Chemical Society.

Low values of ω indicate good electron donor molecules. High values of ω+ are necessary for good electron acceptor molecules. These two quantities refer to charge transfers and not necessarily one electron. These chemical descriptors have been used successfully in different chemical systems.^28−33^ With these parameters, it is possible to determine the electron donor–acceptor map (DAM, see Figure 2).^30^ Systems located down to the left are considered good electron donors, while those situated up to the right are good electron acceptors.

Global softness is also a global response function, which is related to polarizability.^41−43^ The greater the global softness, the greater the polarizability. Global softness (*S*) was obtained as the inverse of hardness (η) by the following equation:
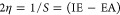
5

## Results and Discussion

To know more about the mechanism of action of psychedelic compounds, we looked at their electron transfer properties. The investigated psychedelic drugs are listed in Figure 1. Mescaline and TMA-2 are similar. The differences are the position of one OCH_3_ group and an extra methyl group close to the amine. 2C-x and DOx families are analogous to those of mescaline. We indicate the compounds with similarities in Figure 1. 2C-D, 2C-E, and 2C-P have methyl, ethyl, and propyl substituents, respectively. The second group of 2C-x has halogens as substituents. The third group presents sulfur and the last is the DOx family with an extra methyl group close to the amine. DOI (2,5-dimethoxy-4-iodoamphetamine) and DOB (dimethoxybromoamphetamine) also have halogens as substituents (I and Cl, respectively). In Figure 1, there is another group of compounds with tryptamine (highlighted in blue). These latter compounds do not have halogens or sulfur atoms. Serotonin and LSD belong to this group of tryptamines.

Contrary to antipsychotic drugs that can be dopamine agonists or antagonists of dopamine and serotonin, psychedelics are all agonists of serotonin.^17^ As previously reported,^29^ the agonists exhibit similar electron transfer properties to those of the neurotransmitter (dopamine or serotonin) and the antagonists present the opposite. The hypothesis here is that the action of hallucinogenic drugs is mediated in part by electron transfer. Since they are all serotonin agonists, they can be expected to have electron transfer properties similar to those of serotonin. Small differences in the electron transfer capacity could be related to disparities in the potency of these drugs.

**Figure 3:**
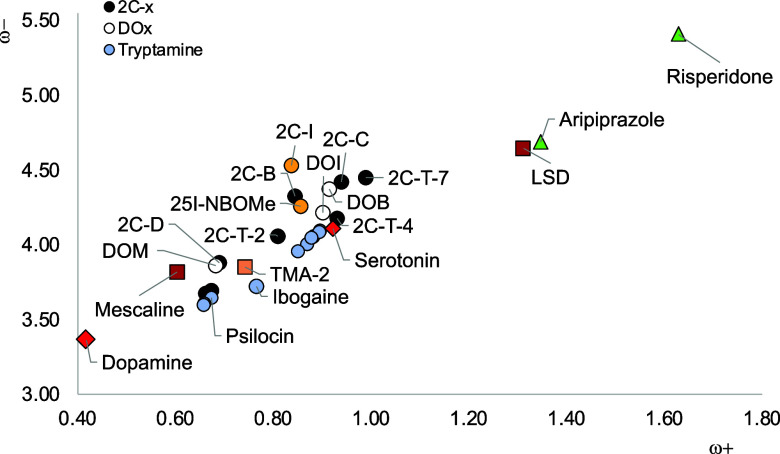
Electron DAM of the studied compounds.

Figure 3 presents the DAM of the systems under investigation. Two antipsychotic drugs are included for comparison: aripiprazole (the partial agonist of dopamine) and risperidone (an antagonist of dopamine). The first thing to notice is that psychedelics, serotonin, and dopamine are all better electron donors and worse electron acceptors than antipsychotics. All are located down to the left with respect of antipsychotics. When the possibility of using hallucinogens to manage psychiatric disorders is kept in mind, it should be important to consider this information. Without seeing antipsychotics, LSD is the best electron acceptor, and dopamine is the best electron donor. Serotonin lies more or less in the middle of the map. Unsurprisingly, since psychedelics are all agonists of serotonin, they have electron transfer properties similar to those of serotonin. They are all located nearby on the map.

Mescaline and LSD are well-known psychedelics with similar purposes of use. Mescaline is the active component of peyote with lower potency than other psychedelics. LSD is a semisynthetic hallucinogenic substance derived from lysergic acid (the natural product found in the parasitic rye fungus). It is one of the most prevalent and potent hallucinogens available and is now investigated as a potential drug for the treatment of various psychiatric disorders. Mescaline has been reported to be approximately 1000–3000 times less potent than LSD.^9^ The potency of these drugs is also determined by the EC50 values. EC50 refers to the effective concentration of a drug that produces a response that is half of the maximum response. The lower the EC50, the greater the potency. Mescaline exhibits an EC50 of 10 μM and an LSD of 7.2 nM for the 5HT_2A_ receptor.^25,26^ Analyzing the electron transfer capacity, the results in Figure 3 indicate that mescaline is a worse electron acceptor than LSD. Similar to mescaline is TMA-2, and according to previous results, it is twice as potent as mescaline.^9^ The results in Figure 3 show that TMA-2 is a better electron acceptor than mescaline. Actually, mescaline is the worst electron acceptor among all of the hallucinogens that we investigate (it is on the left), and it has been reported as one with the lowest potency. DOB and 2C-B have a bromine atom in the structure. The first is ten times more powerful than the second.^10^ From Figure 3, it is possible to notice that DOB is a better electron acceptor than 2C-B (it is on the left).

The first conclusion that arises from these results is that the electron-acceptor capacity is related to the potency of the drugs. Apparently, more potent drugs are better electron acceptors. LSD is one of the most powerful hallucinogenic drugs known, and it is the best electron acceptor. Should this be the case, 2C-x and DOx drugs with halogens or sulfur atoms would be more potent than those that only contain carbon, hydrogen, oxygen, and nitrogen in the formulation. The presence of halogens and sulfur atoms influences the capacity of the electron transfer and makes these compounds better electron acceptors. Those C2-x drugs with methyl, ethyl, and propyl substituents are closer to mescaline and are worse electron acceptors but better electron donors than the other members of the 2C-x family. DOM also has a methyl group as a substituent. A lower potency of these drugs could be expected since they are worse electron acceptors, but this has to be corroborated. 25I-NBOMe and 2CI are two compounds with iodine. It was reported^27^ that 25I-NBOMe is 30-fold more potent at rat 5-HT_2A_ receptors when compared to 2C-I, but the results in Figure 3 indicate that they have similar electron acceptor properties. This is an exception that cannot be explained by the DAM.

**Figure 4:**
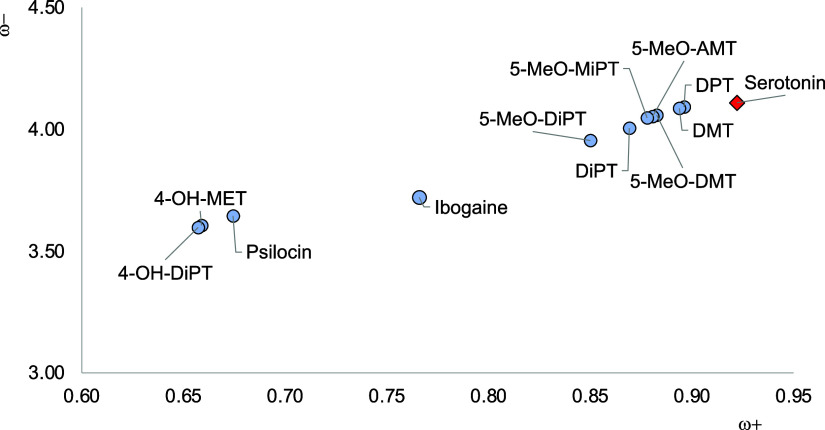
Electron DAM of the studied tryptamines.

All tryptamine derivatives have similar electron transfer properties, close to those of serotonin or mescaline. In general, they are better electron donors than the 2C-x and DOx families (they are located lower on the map). All tryptamine derivatives have only C, H, O, and N atoms in their formulation. Experimental information indicates that tryptamines present differences in potency, but this is difficult to establish since it depends on the routes of administration.^8^ To analyze the tryptamine results in more detail, an amplification of DAM is presented in Figure 4. LSD is not included.

The presence of hydroxyl or methoxy groups at positions 4 and 5, respectively (see Figure 1), was related previously with an increment of the potency of tryptamine derivatives.^8^ DMT (*N*,*N*-dimethyltryptamine), DiPT (diisopropyltryptamine), and DPT (*N*,*N*-dipropyltryptamine) do not have hydroxyl or methoxy groups. They show similar electron acceptor properties to compounds with methoxy groups, and they are better electron acceptors than those with hydroxyl groups (4-OH-MET, 4-OH-DiPT, and psilocin).

According to the results in Figure 4, the compounds with hydroxyl groups are better electron donors and worse electron acceptors than other tryptamines. Following our hypothesis, they should be less potent than the others, but this is not the case. Experimental observations suggest that hydroxyl or methoxy groups increase the potency. However, caution is required before discarding our hypothesis since the potency analysis for tryptamines comes from the doses consumed by users. The lower the dose, the more the power. It is very difficult to determine the potency based on the doses since the effects produced also depend on the particularities of the consumer and the routes of administration. The choice of the measured response is also important and determines the results.^3,5^ This represents problems in determining doses and, therefore, potency. In any case, the electron transfer properties of tryptamines are similar, and the discrepancies in the electron donor–acceptor capacities are smaller than those found for the 2Cx and DOx families. It can be thought that the differences in the potency of these tryptamines are not as high as those with the C2-x and DOx families.

There has been an effort to understand the effects of hallucinogens by considering the binding energies to specific sections of the receptor, but some authors^3^ emphasized that “binding affinity values may not be directly proportional to drug potency”. It was reported that the compounds in Figure 3 exhibit high binding affinities to the receptor, but this does not correlate well with the effects produced by these drugs. The binding affinities, or dissociation constants, reflect chemical/thermodynamic properties of the interaction of the ligand with the receptor, while potency measurements are relative, for example, to the choice of the measured response. The association between the 5-HT_2A_ receptor and the perception-altering properties of psychedelics in humans is well-known, but the relationship with binding affinities is not evident; therefore, there is no specific explanation for the mechanism of action of hallucinogens. Receptor binding is important, but we think something has to happen once the drug interacts with the receptor. Our proposal based on the results reported here is that there is an electron transfer process and this somehow produces hallucinations. The best electron acceptors are also the highest potency psychedelics. The electron-accepting capacity related to potency is something that must be taken into account. There are many other things that must be contemplated to fully understand the mechanism of action of these drugs, such as permeability to the blood–brain barrier, rates of metabolism and elimination, and the formation of active metabolites. The need to investigate all these factors does not invalidate the results of this research. This research can be considered as a model of the first step of the action mechanism.

**Figure 5:**
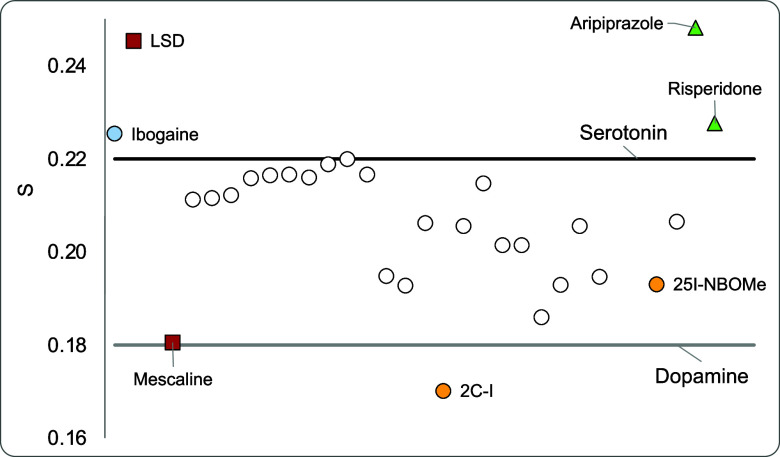
Global softness (*S*).

Many of the well-known empirical chemical concepts, such as hardness (η) and softness (*S*), appear naturally within the DFT framework. These are global parameters that help us to understand the reactivity of the systems. The principle of maximum hardness makes the hardness the most popular since it is related to the stability. In this case, we want to analyze the opposite, i.e., the reactivity. For this reason, we consider global softness as a better descriptor. There have been numerous analytical and numerical evidence, indicating that softness has a closer link with polarizability.^41−43^ In this investigation, global softness is used to characterize the drugs. Figure 5 reports the global softness for all of the compounds under investigation. The two lines represent the values of dopamine and serotonin.

Most of the molecules are between the values of dopamine and serotonin. Within the hallucinogens, LSD is the most potent, and mescaline is less potent. As indicated in Figure 5, the global softness of LSD is higher than that of mescaline. The potency of 25I-NBOMe is greater than that of 2C-I, and the global softness of the former is greater than that of the latter. Apparently, global softness is related to the potency of the drugs. Greater global softness implies greater potency. This makes sense because polarized molecules form dipoles, and dipoles can promote electron transfer. This could be the mechanism of the drug activation.

Electron transfer properties and global softness are receptor-independent properties of the drugs. We do not contemplate the receptor in this investigation, since we consider important to characterize the drugs independently, to have like a fingerprint of these molecules. A good analogy was previously reported,^29^ emphasizing the importance of studying these drugs. The model used was named the model of bulbs and sockets. The bulbs represent the drugs, and the sockets represent the receptor. Some characteristics of bulbs are independent of the sockets (e.g., bulbs can have different voltages). Likewise, certain characteristics of drugs are independent of receptors, such as electron transfer capacity. In this model, the process of recognition of molecules by the receptor in the active sites is represented by sockets and bulbs. The bulbs must fit into the socket, and the receptor must recognize the drug. After this initial stage, something needs to happen to produce the psychedelic effects, just as something needs to happen to turn on light bulbs. Once the molecule binds to the receptor, this research suggests that there is a transfer of electrons that is related to the molecule’s function in producing psychedelic effects. The comparison between drugs shows relative values of electron transfer, regardless of the characteristics of the receptor, and this is useful to characterize the drugs.

## Conclusions

Scientific studies of psychedelic drugs are a recent field of research. Unlike other drugs, for hallucinogens, studies in humans are essential to fully understand the mechanism of action, since it is necessary to discover conscious contents, and this is impossible with animal models. To determine the potency and effectiveness of psychedelic drugs, there are many complications because potency depends on the doses, the personality of the user, the route of administration, and the way of measuring the effects (changes in language, hallucinations, types of ideas, or how long the effects of the drug last). Theoretical studies are also not easy since receptors are complex proteins. Due to all of these difficulties, it is important to characterize the drugs to have something like a fingerprint of the molecules. This will help us to understand the mechanism of action.

In this investigation, we characterize psychedelic drugs using their electron transfer properties. All of these drugs are serotonin agonists and show electron transfer properties similar to those of serotonin, with minor differences that are important. The electron-accepting capacity is directly related to the potency of the drugs. LSD is the most powerful hallucinogen and also the best electron acceptor molecule. Mescaline is one of the least potent drugs and is the worst electron acceptor among all of the psychedelic molecules under study. Another chemical descriptor that correlates well with the potency is global softness. Global softness is directly related to polarizability, and apparently, this is important in explaining drug potency. Molecules with greater global softness also have greater potency. With these two descriptors, we gain information concerning the action mechanism of psychedelic drugs. Several properties are involved in the effects of psychedelic drugs. For future research, it might be appropriate to define schemes with quantitative and qualitative properties, such as the EC50 indicating drug potency. It might be possible to calculate the correlation coefficient that would allow us to provide a range of therapeutic doses for all of the drugs. This possible future work could improve our knowledge of the mechanism of action of psychedelic drugs.
